# Respiratory Morbidity After Repair of Type C Esophageal Atresia with Tracheoesophageal Fistula: A Pediatric Case Series

**DOI:** 10.3390/jcm15176922

**Published:** 2026-09-07

**Authors:** Cristiana Sophia Mihordea, Tudor-Gabriel Neagu, Daniela Pop, Oana Maria Mitrașca, Anca-Valina Gîngă, Edita-Gabriela Ichim, Valentina Sas, Sorin Claudiu Man, Paraschiva Chereches-Panta

**Affiliations:** 1 Faculty of Medicine, “Iuliu Hațieganu” University of Medicine and Pharmacy, 400012 Cluj-Napoca, Romania; neagu.tudorgabriel@elearn.umfcluj.ro (T.-G.N.);; 2 Third Pediatric Clinic, Clinical Hospital for Pediatric Emergencies, 400132 Cluj-Napoca, Romaniapusachereches@umfcluj.ro (P.C.-P.); 3 Third Pediatric Discipline, Department 8, Mother and Child, Faculty of Medicine, “Iuliu Hațieganu” University of Medicine and Pharmacy, 400124 Cluj-Napoca, Romania

**Keywords:** esophageal atresia, tracheoesophageal fistula, pneumonia, recurrent wheezing, asthma

## Abstract

**Background:** Esophageal atresia (EA) is a rare congenital malformation of the foregut, most commonly presenting in association with tracheoesophageal fistula (TEF). Advances in neonatal surgical techniques and intensive care have significantly improved survival rates over recent decades. However, short- and long-term respiratory morbidity remains prevalent, substantially impacting patients’ quality of life. **Methods:** This is a retrospective single-center case series of pediatric patients with repaired EA, with emphasis on the spectrum of pulmonary complications and their clinical management. Six patients with repaired type C EA with TEF and associated respiratory complications were included. All patients were admitted between 2018 and 2026 to the Third Pediatric Clinic, Clinical Hospital for Pediatric Emergencies, Cluj-Napoca, Romania, for the management of respiratory pathology. **Results:** Tracheomalacia was identified in all six patients, and each had experienced at least one episode of lower respiratory tract infection. Reactive airway disease meeting diagnostic criteria for asthma was documented in two patients. One case raised clinical suspicion for a residual or recurrent tracheoesophageal fistula, warranting further diagnostic workup. Gastroesophageal reflux (GER) was present in all patients. Protein-energy malnutrition was identified in two patients (30% of the cohort). **Conclusions:** Surgically repaired EA is frequently associated with considerable chronic respiratory and gastrointestinal morbidity. A structured multidisciplinary approach, including pediatric pulmonology, gastroenterology, surgery and nutrition, alongside systematic long-term follow-up, is essential to optimize outcomes and quality of life in this vulnerable patient population.

## 1. Introduction

Esophageal atresia (EA) with or without tracheoesophageal fistula (TEF) is a congenital anomaly with a prevalence of 2.43 per 10,000 births in Europe [1]. The condition is thought to arise from a defect in septation of the foregut into the trachea and the esophagus at approximately 4 weeks of embryonic development [2]. Prenatal detection of EA continues to pose significant diagnostic challenges, with reported detection rates of only 24% to 32%. The most common sonographic findings include polyhydramnios, a small or absent stomach bubble and a blind-ending dilated proximal esophageal pouch [3,4]. Postnatally, insertion of a nasogastric tube is recommended as a routine diagnostic step in any neonate with suspected EA, as failure to advance the tube beyond the upper esophagus is a key confirmatory finding [5]. Once the diagnosis is confirmed, prompt surgical referral and intervention are warranted.

EA is not just a neonatal surgical emergency but a lifelong condition. Once regarded as a fatal condition, survival rates have increased to >90% as a result of advances in neonatal intensive care and improved surgical approaches [6]. As more patients survive into adolescence and adulthood, however, attention has increasingly shifted toward the long-term aerodigestive morbidity that many of these individuals continue to experience [7]. Chronic respiratory morbidity represents one of the most significant and enduring challenges for EA with TEF survivors. Long-term respiratory complications span the upper and lower respiratory tracts and are closely linked to aspiration and dysphagia. The most frequently reported persistent symptoms include cough (up to 75%), wheezing (up to 40%) and dyspnea (up to 37%) [6].

The pathophysiology is inherently multifactorial and closely intertwined with gastrointestinal dysfunction. Tracheomalacia predisposes patients to respiratory complications, while aspiration due to impaired airway protection reflexes is an underlying contributing mechanism. The relationship between gastroesophageal reflux disease (GERD) and respiratory disease is particularly important. In the presence of GERD and dysphagia, chronic micro-aspiration of gastric contents contributes to airway inflammation, creating a vicious cycle between the digestive and respiratory systems [8].

Despite this, respiratory symptoms are often overlooked compared to digestive complaints, underscoring the need for systematic pulmonary assessment as part of structured, lifelong follow-up. This study aimed to describe the range of pulmonary complications and their clinical management in a case series of pediatric patients with repaired type C EA followed for respiratory pathology at a tertiary pediatric center in Cluj-Napoca, Romania.

## 2. Materials and Methods

This study comprises a retrospective case series focusing on respiratory morbidity following surgical repair of type C EA with TEF.

### 2.1. Patient Selection and Clinical Setting

Over an eight-year period (March 2018–March 2026), 27 cases of esophageal atresia were admitted to the Department of Pediatric Surgery, Clinical Hospital for Pediatric Emergencies, Cluj-Napoca, Romania. Of these, 18 (66.7%) were male and 9 (33.3%) were female, yielding a male-to-female ratio of 2:1. Patients were identified through the hospital’s electronic data system using International Classification of Diseases, 10th Revision (ICD-10) diagnostic codes.

Six patients with repaired Type C EA were included in the retrospective case series. Inclusion was based on admission to the Third Pediatric Clinic, Emergency Clinical Hospital for Children, Cluj-Napoca, Romania, between 2018 and 2026, for the evaluation and management of respiratory pathology. Patients admitted for indications other than respiratory pathology were excluded.

### 2.2. Data Collection

The following data were retrospectively extracted from medical records: (1) demographic characteristics, including age and gender; (2) EA/TEF type, mode of surgical repair and postoperative surgical complications; (3) respiratory symptoms and diagnoses, including laryngitis, wheezing, asthma and recurrent respiratory infections; (4) esophageal symptoms, including pyrosis, acid regurgitation and dysphagia to solids or liquids; (5) nutritional status and presence of malnutrition; (6) treatment; and (7) results of diagnostic evaluations, including barium swallow, upper gastrointestinal endoscopy, tracheobronchoscopy, chest X-ray, computed tomography (CT) of the chest, magnetic resonance imaging (MRI) of the chest and pulmonary function testing.

### 2.3. Definitions

Laryngitis was defined as inflammation of the larynx characterized by hoarseness or dysphonia, with or without odynophagia, in the absence of an alternative diagnosis.

Bronchiolitis was defined as an episode of acute lower respiratory tract infection in an infant, characterized by respiratory distress, tachypnea and diffuse expiratory wheezing or crackles on auscultation, consistent with viral-induced small airway inflammation.

Bronchitis was defined as an acute respiratory illness characterized by cough lasting more than five days, with or without sputum production, in the absence of radiographic evidence of pneumonia or an alternative underlying diagnosis.

Recurrent pneumonia was defined as two or more episodes of pneumonia within one year or three or more episodes at any time, each separated by a period of complete radiographic clearance, confirmed by a chest radiograph in conjunction with consistent clinical findings.

Asthma diagnosis was based on the Global Initiative for Asthma (GINA) criteria, requiring a history of typical respiratory symptoms: episodic dyspnea, cough and chest tightness, with objective evidence of variable expiratory airflow limitation, wheezing on physical examination, suggestive spirometry findings and a positive response to beta-2 agonists [9].

Gastroesophageal reflux (GER) was defined, in accordance with the European Society for Pediatric Gastroenterology, Hepatology, and Nutrition/North American Society for Pediatric Gastroenterology, Hepatology, and Nutrition (ESPGHAN/NASPGHAN) guidelines, as the passage of gastric contents into the esophagus, while GERD was considered when symptoms affected daily functioning and/or in the presence of complications [10].

Malnutrition, defined per Academy of Nutrition and Dietetics/American Society for Parenteral and Enteral Nutrition (AND/ASPEN) criteria as a weight-for-age or weight-for-length/height Z-score between −1 and −1.9 standard deviation for mild malnutrition, −2 to −2.9 for moderate malnutrition and ≤−3 for severe malnutrition, was used as a marker of growth faltering and feeding difficulty in the context of a structural condition [11]. Malnutrition status was determined based on a single anthropometric measurement obtained at the time of presentation. Anthropometric measurements were referenced against the CDC growth charts.

Therapeutic decisions reflected individualized clinical management and local practice. It should not be interpreted as standardized treatment recommendations.

The research was conducted in accordance with the Declaration of Helsinki. Written informed consent for the use of clinical data was obtained from the legal guardian of all patients involved in the study as part of the general protocol for admission to the clinical ward. Ethical review and approval from the ethics committee were waived for this study due to the retrospective and anonymized nature of the study.

## 3. Case Reports

We present six cases of repaired EA/TEF in which chronic respiratory morbidity emerged as a defining feature of the long-term clinical course, illustrating the heterogeneity of its presentation, the interplay with underlying aerodigestive dysfunction and the challenges of diagnosis and management across different stages of childhood.

### 3.1. Case 1, B.L.

A 1-year-old boy was admitted to the hospital, presenting with a productive cough, fever and difficulty breathing. The patient is the first child of the family, born via full-term cesarean section, with an Apgar score of 9 and weighing 3100 g at birth. He had a history of Gross type C EA with distal TEF and underwent surgical repair on day 2 of life, complicated by aspiration pneumonia. During hospitalization, the patient underwent two bronchoscopies that revealed tracheomalacia. Cystic fibrosis was suspected, and genetic testing was performed, yielding a negative result. The patient had normal growth and development. His medical history also revealed recurrent bronchiolitis—3 episodes—and GERD—diagnosed clinically, with supportive findings on barium swallow. The patient had chronic exposure to secondhand smoke, as the mother was an active smoker.

**Figure 1:**
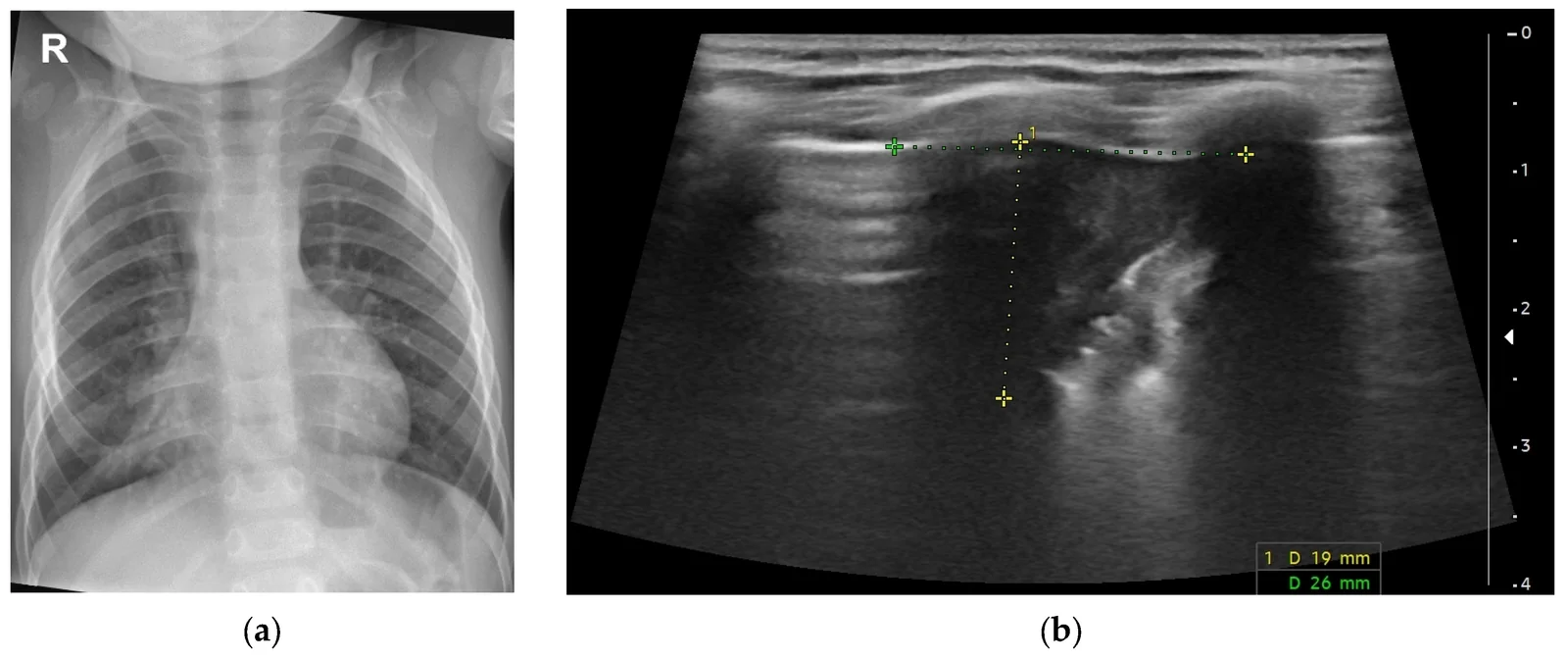
(**a**) Posteroanterior chest radiograph of a 1-year-old male with right middle lobe pneumonia. (**b**) Lung ultrasound of the same patient reveals pulmonary consolidation, pleural irregularity and an adjacent pleural effusion.

Six days before admission, the patient presented with cough and fever (38 °C) and was diagnosed with acute laryngitis and bilateral serous otitis media. Persistent fever and cough were subsequently associated with progressive respiratory distress over the next three days. He presented to the Emergency Pediatric Department with dysphonia, abdominal breathing, subcostal and suprasternal retractions, a respiratory rate of 52 breaths per minute, oxygen saturation of 87%, vesicular breathing with prolonged expiratory phase and crackles in the right lung. Blood analysis showed leukocytosis (16,000/µL) with neutrophilia (9800/µL) and C-reactive protein (CRP) 1.46 mg/dL (normal level < 0.5 mg/dL). Blood gas analysis indicated decompensated respiratory acidosis. The chest X-ray revealed mild pulmonary hyperinflation and a right paracardiac opacity in the middle lobe, obscuring the cardiac contour. A lung ultrasound confirmed the diagnosis of pulmonary consolidation measuring 26/19 mm with an internal air bronchogram (Figure 1).

After initial assessment, he was diagnosed with right middle lobe pneumonia, severe recurrent wheezing, type 2 acute respiratory failure and decompensated respiratory acidosis.

Upon admission, oxygen therapy was initiated on high-flow nasal cannula. Oxygen therapy was maintained for a total of 8 days. On day 2, respiratory syncytial virus (RSV) was detected, and Streptococcus pneumoniae was identified by Multiplex PCR Panel; the latter was interpreted in the context of clinical and radiographic pneumonia. During hospitalization, the patient received antibiotic therapy with a third-generation cephalosporin for 7 days, corticosteroid therapy, bronchodilator therapy (short-acting beta-2 agonist and anticholinergic agent) and intravenous fluid resuscitation. The patient demonstrated a favorable clinical evolution.

The final diagnoses were pneumococcal pneumonia, severe RSV infection, type 2 acute respiratory failure, decompensated respiratory acidosis, viral-induced recurrent wheezing and surgically corrected type C EA with distal TEF and tracheomalacia.

### 3.2. Case 2, J.E.

A 2-year-old boy was admitted to the hospital presenting with dysphonia, difficulty breathing and stridor. He is the second child, born by cesarean section at 35 weeks. In the third trimester of pregnancy, polyhydramnios was reported. The patient weighed 2600 g at birth with an Apgar score of 8. He was diagnosed with Gross type C EA with distal TEF and underwent surgical repair on day 2 of life. After surgery, he developed right upper lobe pneumonia. The genetic testing for cystic fibrosis was negative. An echocardiogram showed a patent foramen ovale, a persistent ductus arteriosus and mild physiological pulmonary hypertension. He was also clinically diagnosed with GERD.

His past medical history also revealed two pneumonias with acute respiratory failure, four episodes of viral-induced wheezing, acute subglottic edematous laryngitis and suspected aspiration of a foreign body, for which resuscitation maneuvers were performed. Bronchoscopy ruled out the suspicion of foreign body aspiration and found a slight degree of tracheomalacia of the posterior tracheal wall, a punctiform invagination of the mucosa at the same level and diffuse edema and congestion of the tracheal mucosa. The patient had a history of passive smoke exposure from the father.

The current episode had a sudden onset of dyspnea, stridor and dysphonia. The patient’s father administered inhaled short-acting beta-2 agonist and inhaled corticosteroid therapy at home, with slight improvement in respiratory distress. The following day, respiratory distress persisted, accompanied by intercostal retractions, prompting presentation to the Pediatric Emergency Department. Clinical examination revealed stridor, bark-like cough, abdominal breathing, peripheral oxygen saturation of 97% and a respiratory rate of 27 breaths/min. Blood investigations demonstrated lymphomonocytosis (leukocytes 10,150/µL, lymphocytes 5005/µL, monocytes 2040/µL) and CRP 0.27 mg/dL.

A diagnosis of moderate viral croup was established. Inhaled epinephrine was administered, associated with inhaled corticosteroid therapy and systemic corticosteroid therapy. During hospitalization, the clinical condition improved within 24 h.

### 3.3. Case 3, B.I.

A 10-year-old girl was admitted to the hospital presenting with a persistent cough and difficulty breathing. She is the second child, born via vaginal delivery at 40 weeks. The patient weighed 3100 g at birth with an Apgar score of 8/9. She was diagnosed with Gross type C EA with distal TEF and underwent surgery on the first day of life. After surgery, she developed tracheomalacia. The patient’s immunization history is limited to the Bacillus Calmette–Guérin vaccine. The parents declined additional vaccinations. Family history is notable for two younger siblings with atopic dermatitis and one younger sibling with asthma.

Her past medical history revealed multiple respiratory complications. From the age of 4 months, she experienced multiple episodes of viral-induced wheezing. Since the age of 5 years, she has been followed at our clinic for partially controlled asthma and has been receiving controller therapy with an inhaled corticosteroid/long-acting β2-agonist combination. Despite controller treatment, she experienced approximately four moderate exacerbations annually. Her most recent spirometry revealed moderate obstructive dysfunction, with a decrease in Forced Expiratory Volume in 1 s (FEV1) to 0.69 L (50% of predicted value) and a significant bronchodilator response following salbutamol administration, with FEV1 increasing to 1.14 L. Immunological work-up was consistent with a Type 2-low asthma phenotype, showing normal eosinophil count, normal total IgE and negative specific IgE testing. To exclude cystic fibrosis, a sweat chloride test was performed at age 8, which returned negative. In the last 5 years, she was diagnosed with nine episodes of acute pneumonia, five of which were complicated by acute respiratory failure. The patient also presented with gastric comorbidities, including a hiatal hernia and secondary GERD with reflux esophagitis, Los Angeles class B, diagnosed on upper gastrointestinal endoscopy and managed with daily proton pump inhibitor (PPI) therapy. She also has a partial fusion of the posterior portions of the fourth and fifth ribs on the right.

**Figure 2:**
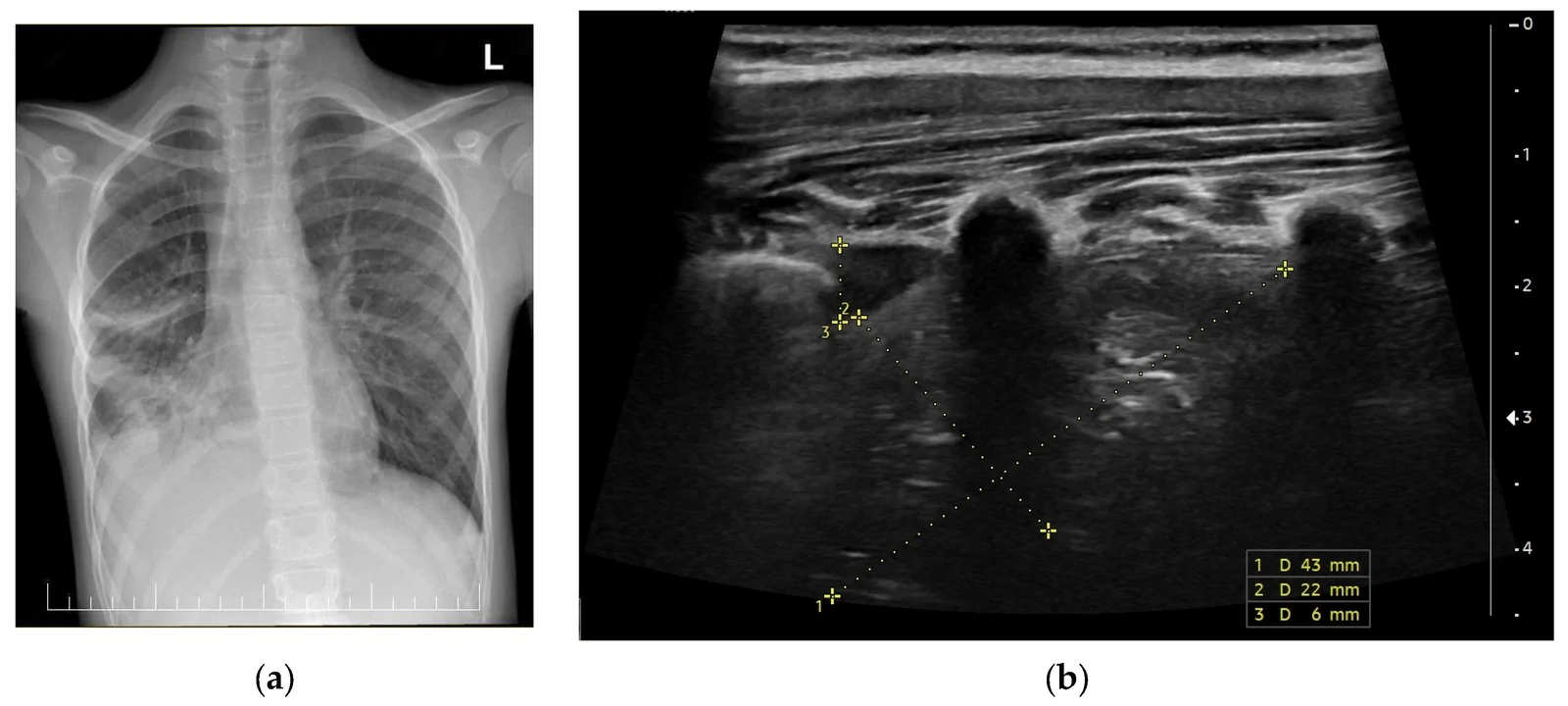
(**a**) Posteroanterior chest radiograph of a 10-year-old female demonstrating bilateral pneumonia with right-sided pleural effusion. (**b**) Lung ultrasound of the same patient reveals pulmonary consolidation and adjacent pleural effusion.

The current episode started suddenly with dry cough and high fever (39.4 °C). The patient’s mother initiated the asthma exacerbation protocol at home. After 4 days without improvement, the patient was brought to the Pediatric Emergency Department. On clinical examination, signs of respiratory distress were present with peripheral oxygen saturation of 90%. Auscultation revealed bilateral basal crackles and wheezing. Anthropometric evaluation revealed a weight of 21 kg (9th percentile, Z-score = −1.37), height of 124 cm (24th percentile, Z-score = −0.7) and BMI of 13.7 kg/m^2^ (6th percentile, Z-score = −1.54), consistent with mild malnutrition. Blood investigations demonstrated neutrophilia and elevated CRP. Chest radiograph was interpreted as bilateral pneumonia complicated by right parapneumonic pleural effusion. The diagnosis was confirmed by lung ultrasound, which revealed pulmonary consolidation measuring 43/22 mm with an internal air bronchogram and an adjacent pleural effusion (Figure 2).

During hospitalization, the patient received oxygen via a simple face mask for 4 days, double antibiotic therapy combining a third-generation cephalosporin and a glycopeptide antibiotic, inhaled bronchodilator therapy with a short-acting beta-2 agonist and an anticholinergic agent and systemic corticosteroid therapy for 5 days. The patient’s clinical condition gradually improved during the first 4 days of hospitalization. Given persistent respiratory distress, a methylxanthine bronchodilator was added for 36 h on continuous infusion, as well as an intravenous magnesium sulfate bolus. Maintenance therapy was continued with inhaled corticosteroid/long-acting beta-2 agonist combination therapy, supplemented with a leukotriene receptor antagonist.

The final diagnoses included partially controlled asthma with a severe exacerbation, acute bilateral pneumonia, type 1 acute respiratory failure, hiatal hernia, GERD with Los Angeles grade B reflux esophagitis, mild malnutrition, repaired type C EA with distal TEF and tracheomalacia.

### 3.4. Case 4, B.V.

A 6-year-old boy presented with persistent cough. He is the first child, born from an uncomplicated pregnancy at 36 weeks of gestation via vaginal delivery, with a birth weight of 2700 g and an Apgar score of 7/9. He was diagnosed with Gross type C EA with distal TEF, which was surgically repaired on the first day of life.

His medical history is notable for several respiratory complications during infancy and early childhood. At 2 months of age, he developed aspiration pneumonia associated with acute respiratory failure. Subsequent investigations revealed tracheomalacia, confirmed by bronchoscopy. He was clinically diagnosed with GERD. The patient experienced multiple respiratory infections, including three episodes of pneumonia, bronchiolitis, recurrent laryngitis and influenza A infection. In 2024, he presented with recurrent monthly bronchial obstruction episodes, leading to the diagnosis of partially controlled asthma. The patient also has associated conditions, including a patent foramen ovale and allergic rhinitis. Family history is notable for allergic asthma in the maternal grandmother and autoimmune thyroid disease in the mother.

The current episode started several days before admission with a dry cough that progressively became productive, nasal discharge and worsening respiratory symptoms. Despite home treatment with inhaled bronchodilators and inhaled corticosteroids, the cough became more frequent, prompting medical evaluation. The patient also received oral corticosteroids, with only partial improvement.

On admission, the patient was afebrile and had a slightly altered general condition. Clinical examination revealed a productive cough, a respiratory rate of 26 breaths per minute and oxygen saturation of 97–98% in room air. Pulmonary auscultation revealed a prolonged expiratory phase with bilateral wheezing, rhonchi and coarse crackles. Cardiac examination was normal, with regular heart sounds and no murmurs. Spirometry performed during hospitalization indicated moderate obstructive ventilatory dysfunction. Baseline FEV1 was 0.67 L (57% of predictive value). After bronchodilator administration, FEV1 increased to 0.92 L (+0.25 L, +37%), indicating a significant bronchodilator response.

Imaging studies were also performed during the admission.

**Figure 3:**
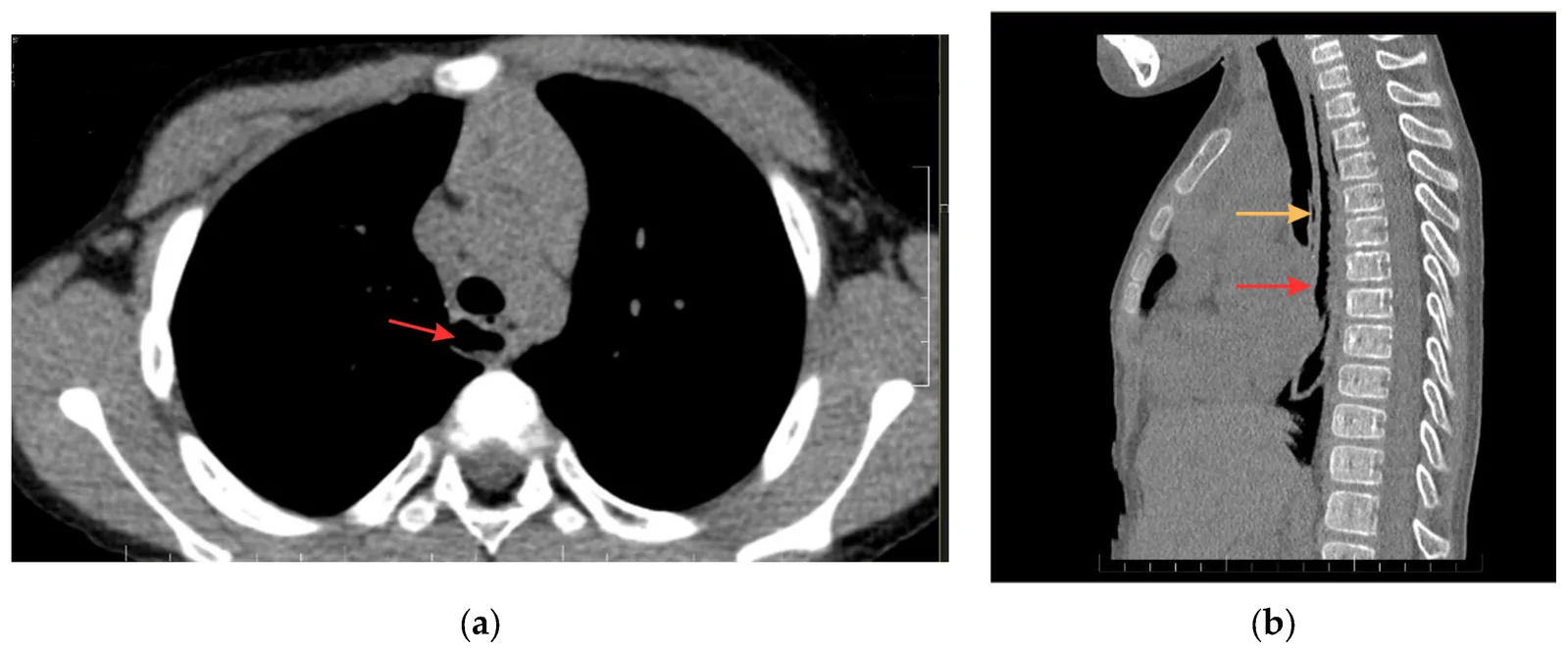
Thoracic CT scan in a 6-year-old boy showing esophageal dilatation (red arrows) and a posterior tracheal air-filled tract (yellow arrow) suspicious for recurrent or residual tracheoesophageal fistula. (**a**) Transverse plane; (**b**) sagittal plane.

Thoracic CT revealed esophageal ectasia with a maximum diameter of 14.5 mm and a hydroaeric level. Additionally, a 24 mm vertical air-filled tract (1.5 mm diameter) located between the trachea and esophagus at the T3–T5 level was identified, communicating with the posterior tracheal wall but without clear communication with the esophageal lumen (Figure 3). These findings raised the suspicion of a possible residual or recurrent TEF. Bronchoscopic confirmation was recommended but was not available at the time of manuscript preparation.

**Figure 4:**
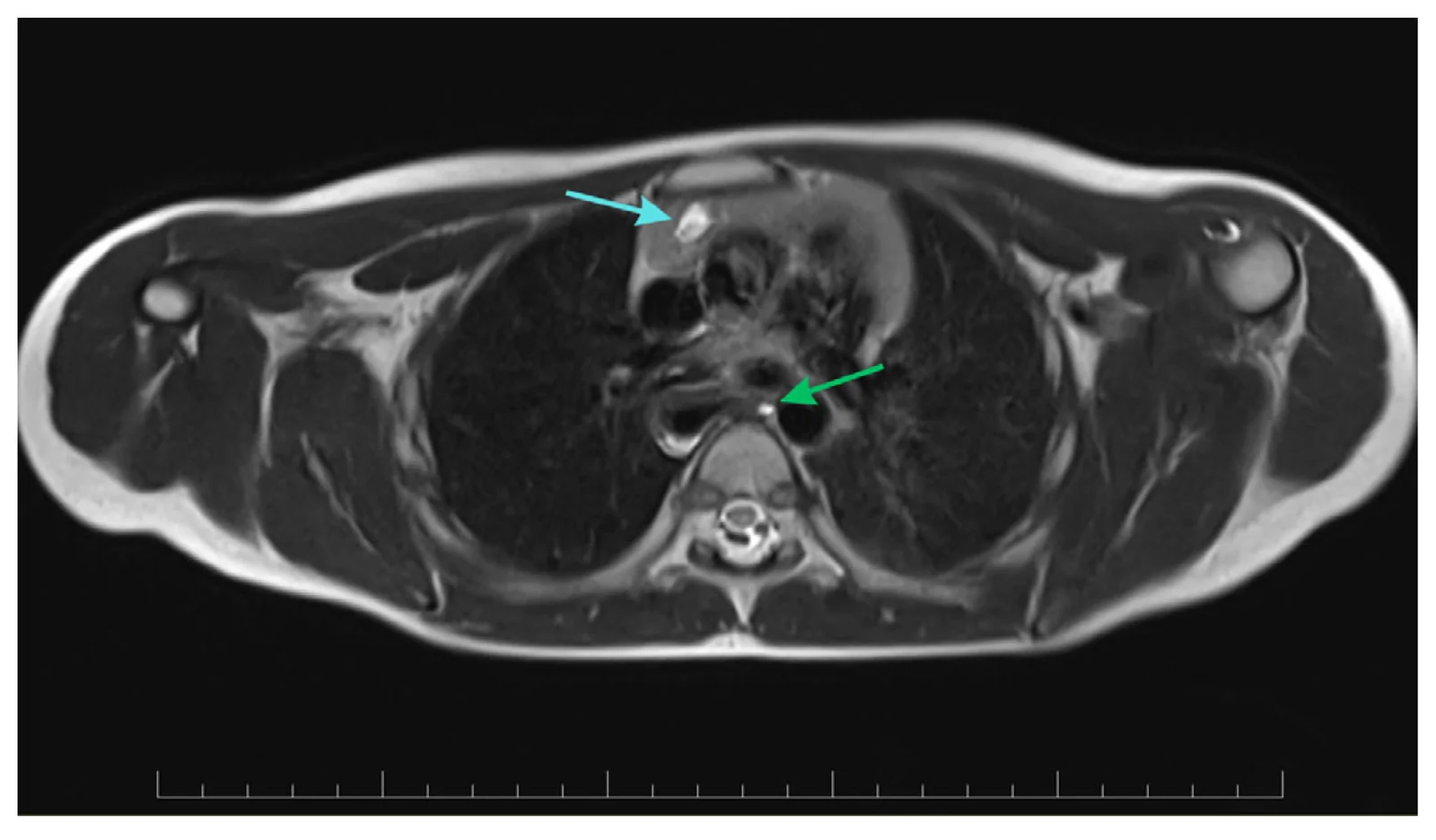
Thoracic MRI of a 6-year-old male, demonstrating a septated cystic formation in the right thymic region (blue arrow) suggestive of mediastinal lymphangioma, diffuse esophageal dilatation along its entire length and a small cystic image (green arrow) adjacent to the esophageal wall.

Thoracic MRI confirmed septated cystic formations in the right thymic region, suggestive of a mediastinal lymphangioma, measuring up to 48.5 mm in length. The esophagus appeared dilated along its entire length, mostly in the upper two-thirds, containing predominantly air. A small cystic image adjacent to the esophageal wall suggested a possible postoperative esophageal diverticulum (Figure 4).

During hospitalization, the patient received oral corticosteroid therapy, inhaled short-acting beta-2 agonist, inhaled anticholinergic therapy, macrolide antibiotic therapy and mucolytic therapy. Maintenance therapy with inhaled corticosteroid/long-acting beta-2 agonist combination therapy was continued, along with antihistamine therapy. The clinical evolution was favorable, with a progressive reduction in cough and respiratory symptoms.

### 3.5. Case 5, H.R.

An 11-month-old boy was admitted to the hospital presenting with a productive cough, serous rhinorrhea and psychomotor agitation. The patient is the third child, born via cesarean section at 41 weeks of gestation, with a birth weight of 3270 g and an Apgar score of 9/10. The patient was diagnosed with Gross type C EA with distal TEF. He underwent surgical repair on day 2 of life, complicated by left pneumonia, left pulmonary atelectasis, pulmonary hemorrhage and tracheo-broncho-laryngomalacia. His past history also revealed GERD with esophagitis, confirmed endoscopically, acute laryngitis, acute pneumonia, pulmonary hemorrhage and selective IgA deficiency.

**Figure 5:**
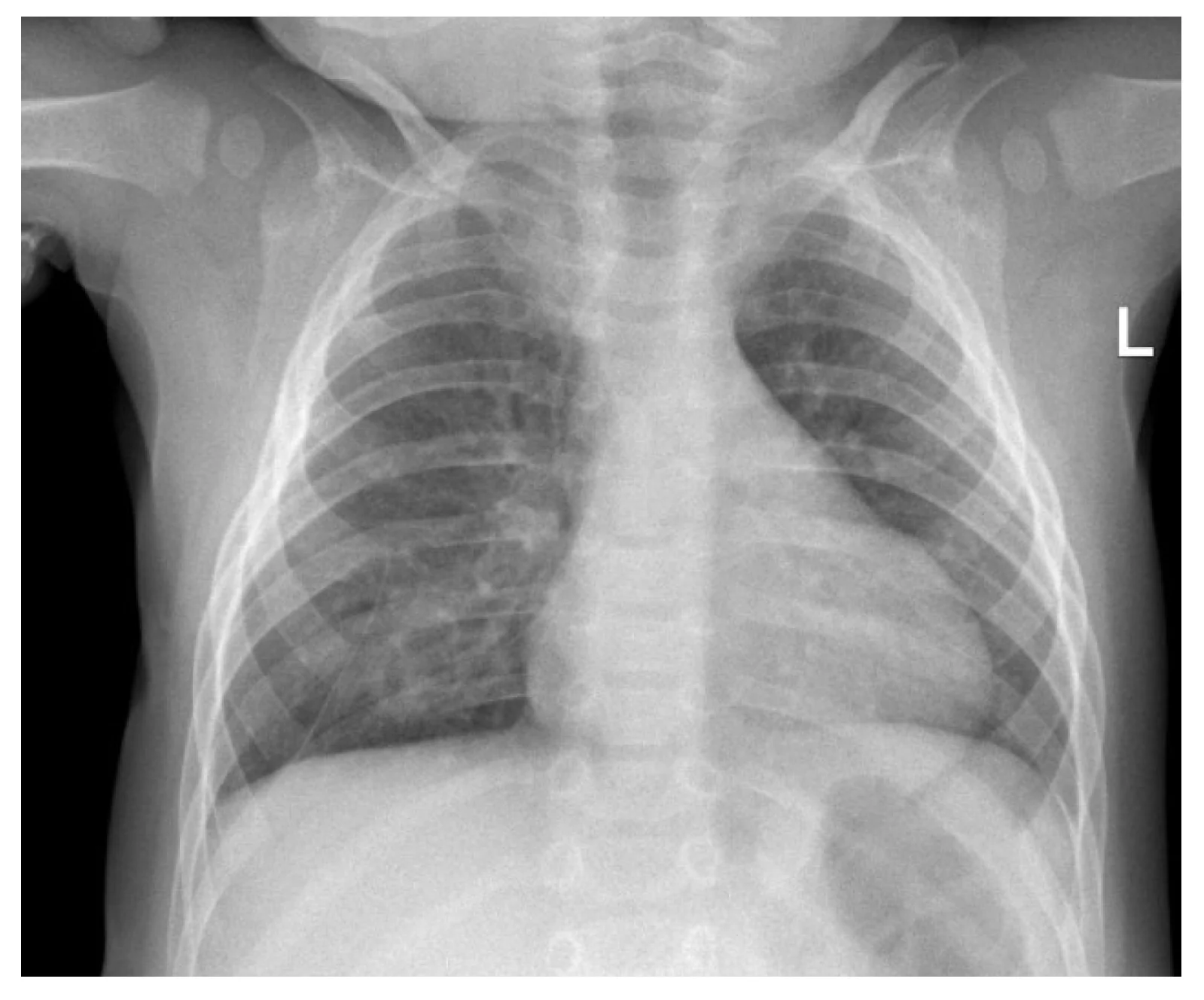
Posteroanterior chest radiograph of an 11-month-old male with acute right-sided pneumonia.

The current illness reportedly began suddenly one day prior with psychomotor agitation. During its course, it was associated with serous rhinorrhea, a productive cough and difficulty breathing, which prompted presentation to the Pediatric Emergency Department. Clinical exam revealed a productive cough, mild subcostal retractions, a respiratory rate of 30 breaths per minute, harsh vesicular breathing with prolonged expiration, disseminated sibilant and rhonchi rales and oxygen saturation of 96% in room air. Laboratory tests revealed leukocytosis with neutrophilia, minimum inflammatory syndrome with a CRP of 1.76 mg/dL and compensated metabolic acidosis. Chest X-ray revealed clouding of the right infrahilar and supradiaphragmatic pulmonary parenchyma and a thickened right perihilar interstitium (Figure 5). The diagnosis was acute right-sided pneumonia, and the patient was redirected to our clinic for monitoring and treatment.

The patient received intravenous antibiotic therapy with a third-generation cephalosporin, intravenous corticosteroid therapy and inhaled bronchodilator therapy combining a short-acting beta-2 agonist and an inhaled corticosteroid. The clinical course was favorable.

### 3.6. Case 6, S.T.

A 6-week-old boy was admitted to our ward for cough, perioral cyanosis, vomiting and difficulty breathing after meals.

At birth, he was diagnosed with Gross type C EA with distal TEF. Surgery was performed on the second day of life. He was born at 37 weeks of gestation by Cesarean section. His birth weight was 3600 g, and his length was 51 cm. Apgar score was 8. At birth, he was also diagnosed with tracheomalacia and a ventricular septal defect with left-to-right shunt.

He developed a productive cough 2 days before admission. This cough was associated with vomiting and difficulty breathing after feeding. He had no fever. The mother reported noisy breathing. On the day of admission, he presented with perioral cyanosis. Upon admission, he was in good general condition. His weight was 3800 g (percentile 7, Z-score = −1.5), length was 55 cm (percentile 30, Z-score = −0.52), and his weight-for-length percentile was 1 (Z-score = −2.17). The respiratory rate was 36 breaths per minute, heart rate was 120 beats per minute, and oxygen saturation was 98%. Breath sounds were diminished on the right lower side of the thorax. An X-ray in the emergency department showed a small right infrahilar area of consolidation. Leucocyte count was normal (11,600/μL), CRP 0.2 mg/dL. He was diagnosed with right acute pneumonia. On the second day of admission, he had an episode of apnea during feeding. This was accompanied by perioral cyanosis and decreased oxygen saturation (70%). We suspected that the TEF was not completely resolved or that he was aspirating gastric contents due to GER. An esophagogram with contrast showed no TEF. He was treated for 7 days with a third-generation cephalosporin. The mother was advised to thicken the milk formula with carob flour. At one-month follow-up, the evolution was good. He had no more vomiting, cyanosis, or cough and gained 800 g in weight.

## 4. Summary of Cases

The respiratory complications are summarized in Table 1. All six of our patients have tracheomalacia. All have had repeated episodes of upper respiratory tract infection, and four have had at least one episode of acute laryngitis. Two of the patients met the clinical and functional criteria for a diagnosis of asthma.

**Figure 6:**
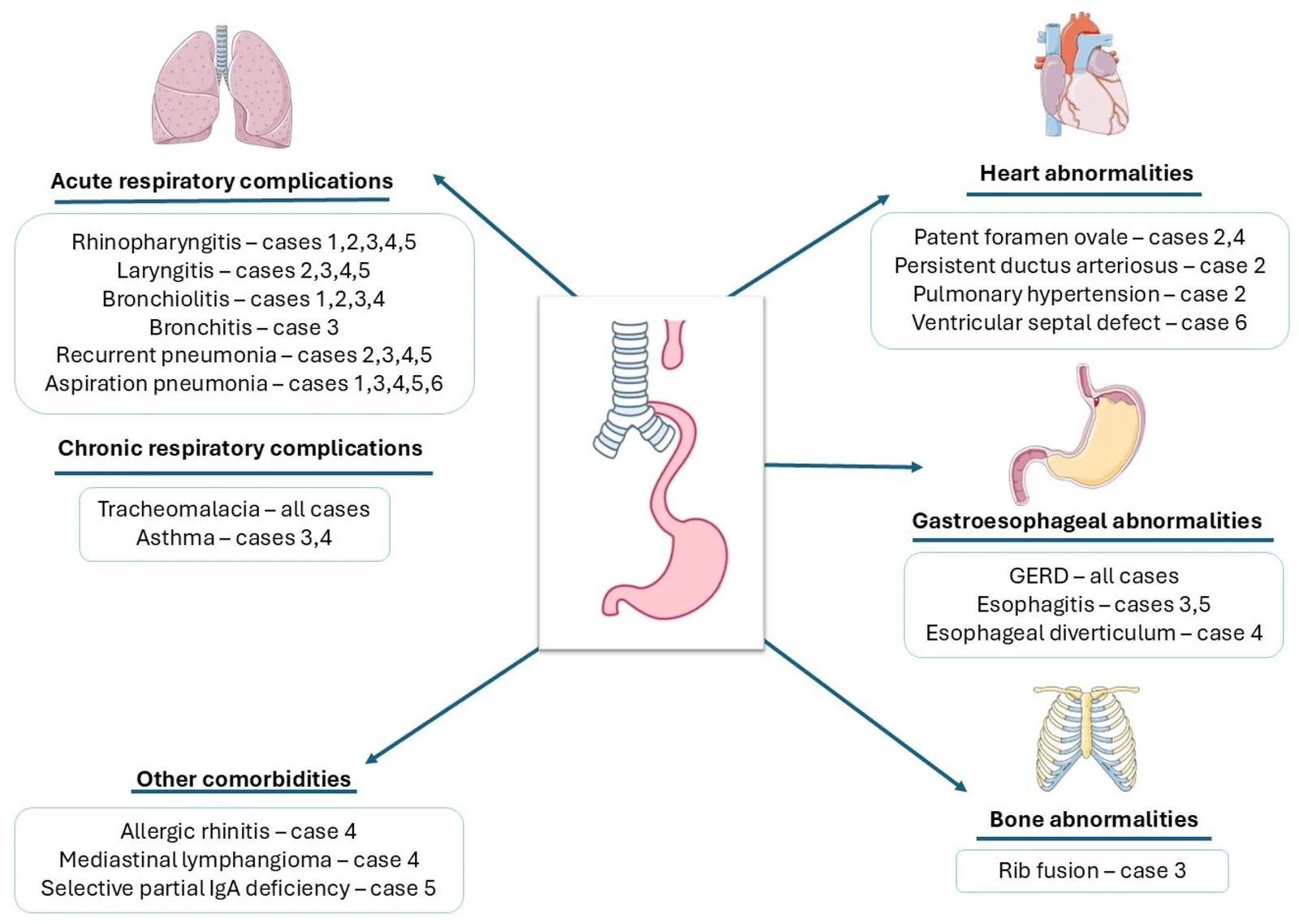
Complication and comorbidity overview. Adapted from Servier Medical Art (https://smart.servier.com), licensed under CC BY 4.0 (https://creativecommons.org/licenses/by/4.0/) accessed on 10 May 2026.

Among the comorbidities, we report congenital heart defects in three of the six patients and a rib anomaly in one case (Figure 6). Gastroesophageal reflux was present in all patients.

## 5. Discussion

**Figure 7:**
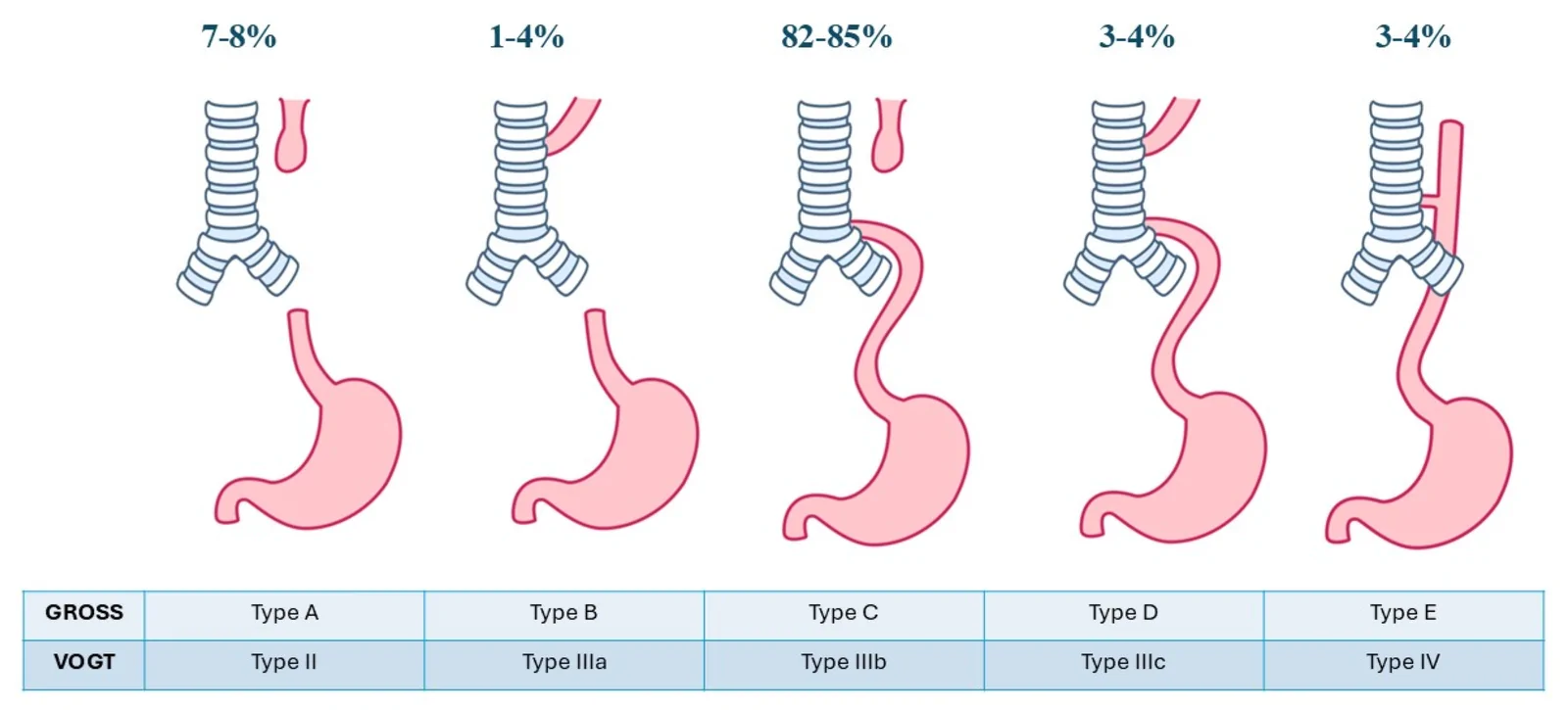
Classification of esophageal atresia (EA). Values above show the frequency of each type.

There are two main classification systems available, Gross and Vogt, that define five subtypes of EA based on the presence and/or placement of the TEF (Figure 7). The most common variant (82–85%) is Gross type C or Vogt type IIIB, also known as EA with distal TEF [6]. Consistent with this epidemiological pattern, all six patients in the present series presented with this subtype.

Five of the six patients in this case series are male. Male predominance has been reported in the literature for EA. For instance, a study published in 2012, which included 1222 patients from 23 European registries, found a male:female ratio of 1:0.74 [12]. However, a 30-year population-based study from Croatia found no statistically significant sex difference, with a male-to-female ratio of 1:1.2 [1].

EA/TEF is linked in about 50% of cases with other malformations, such as VACTERL association—a group of congenital anomalies that include vertebral defects, anal atresia, cardiac defects, TEF, renal abnormalities and limb anomalies [1]. Other associations include CHARGE syndrome, POTTER syndrome and SCHISIS syndrome [13].

The patients demonstrated multiple respiratory complications commonly associated with this condition, including laryngitis, bronchiolitis, bronchitis, recurrent pneumonia and asthma, reflecting the well-documented susceptibility of this population to both structural and inflammatory pulmonary pathology. The frequency and severity of these findings underscore the significant long-term respiratory burden carried by children following surgical repair of esophageal atresia, warranting close multidisciplinary follow-up. However, it is important to note that the six patients described in this series were selected from a larger cohort of children with repaired EA specifically because they presented with associated respiratory morbidity. This selection criterion means that the findings reported here reflect the clinical profile of a subgroup with respiratory symptoms severe enough to warrant closer evaluation, rather than the expected frequency of these conditions among children with repaired EA with TEF in general.

Short-term pulmonary impairments are related to surgical complications, such as anastomotic leaks, stricture and recurrence of fistula.

Anastomotic leakage affects approximately 10–25% of patients after surgery [14]. Predisposing factors include tension of the anastomosis, long procedure time, stage repair surgery, as well as prematurity and low birth weight [14,15]. Leaks represent major risk factors for life-threatening complications, including sepsis, pneumonia, atelectasis, pleural effusion and tension pneumothorax [16]. It is also associated with an increased risk of recurrent TEF and development of strictures [17].

Anastomotic strictures are common esophageal complications, occurring in 30–50% of cases. Manifestations of anastomotic stricture include dysphagia, regurgitation, aspiration pneumonia and chronic pulmonary infections [16]. In most cases, the treatment consists of repeated dilatations with bougies or hydrostatic balloons [17]. For recurrent or refractory strictures, adjuvant methods may be used—endoscopic steroid injection, systemic steroid administration or topical application of mitomycin C [16].

Recurrent TEF affects about 5–10% of patients with EA and typically develops within 2–4 months but can remain undiagnosed for a long period of time [16]. Its symptoms are not specific and often mimic those of tracheomalacia and GER, which can make diagnosis difficult. On chest X-ray, it might appear as air in the mediastinum, but an esophagram with contrast and a laryngotracheobronchoscopy should be performed [6]. Management involves complex revision surgery and carries a high risk of further recurrence [18]. Thoracic CT findings in Case 4 raised the suspicion of a residual or recurrent TEF. An esophagram with contrast was subsequently performed and did not reveal evidence of a fistula. Given the known limited sensitivity of the esophagram for small fistulas, bronchoscopic confirmation was recommended as the next diagnostic step. Definitive endoscopic confirmation was therefore not available at the time of manuscript preparation, and this case remains under active surveillance within our multidisciplinary team. The respiratory findings in this patient should be interpreted with this uncertainty in mind, as an unconfirmed fistula could independently contribute to recurrent aspiration and respiratory symptoms.

Long-term respiratory symptoms are common in patients born with EA. A large study published in 2025, which included 5052 EA/TEF cases from 2004–2024, found that EA/TEF survivors had significantly higher rates of asthma (28% vs. 9%, *p* < 0.0001), tracheomalacia (29% vs. 0%) and pneumonia (31% vs. 11%) compared to matched controls. In addition, the gap in combined aerodigestive morbidity rates between the EA/TEF cohort and the control group widened progressively over long-term follow-up (*p* < 0.001) [19].

Tracheomalacia is characterized by weakness of the tracheal wall, which may affect the entire trachea or be limited to a specific segment. This condition is frequently associated with EA—up to 78%. Sometimes bronchomalacia is present as well. Normally, the tracheal lumen expands during inspiration and narrows during expiration, as airway diameter depends on the balance between intrathoracic and intraluminal pressures. Tracheomalacia leads to excessive tracheal collapsibility, exaggerating the normal narrowing that occurs during expiration [20]. This condition can present with symptoms such as expiratory stridor, persistent cough and reduced exercise tolerance. It is frequently mistaken for asthma or other reactive airway conditions. Tracheomalacia has long been underdiagnosed, even though its management differs substantially from standard asthma treatment and may involve both medical and surgical interventions [19]. In more severe cases, significant airway obstruction can develop, particularly when intrathoracic pressure rises, such as during coughing, crying or forced expiration. This can cause “dying spells”, also known as “apneic spells” or “blue spells” [20,21]. Tracheomalacia is also associated with inefficient cough and mucus buildup, due to abnormal mucociliary clearance, leading to persistent or recurrent respiratory infections [21]. In our study, all six patients had tracheomalacia and experienced at least one episode of pneumonia. Given the co-occurrence of other potential contributors to respiratory morbidity in these patients—including GERD, possible aspiration, wheezing or asthma—this case series cannot establish a causal or independent role for tracheomalacia in pneumonia risk. Nonetheless, the consistent presence of tracheomalacia across all six cases suggests it may be a relevant associated finding warranting further investigation in larger, controlled cohorts.

Recurrent pneumonia and pulmonary infections represent one of the most prevalent long-term complications following surgical repair of EA. In a large retrospective cohort of 105 children with surgically corrected EA, 66% reported lower respiratory symptoms, with recurrent pneumonia (33%) and wheezing (31%) being the most frequently reported [22]. The underlying mechanisms are multifactorial and may be further compounded by individual risk factors. In our series, risk factors for recurrent respiratory infections were identified in five of the six patients: passive exposure to tobacco smoke (Cases 1 and 2), incomplete vaccination (Case 3), atopic family history (Cases 3 and 4) and IgA deficiency (Case 5). Three of the six patients developed pneumonia in the early postoperative period, while two patients had multiple episodes of pneumonia throughout their lives. Pneumonia is a frequent reason for readmission after EA repair. In a retrospective study including 105 patients, Porcaro et al. reported a 33% incidence of recurrent pneumonia, with a mean age of onset of 2.2 ± 2.5 years [22]. Pneumonia in patients with EA may also develop secondary to aspiration, as abnormal swallowing, GERD and recurrence of TEF at the surgical repair site can all precipitate aspiration and recurrent lower respiratory tract infections. In our cohort, aspiration was clinically suspected in five of the six patients on the basis of feeding-related coughing or choking and radiographic findings consistent with aspiration, but it was not objectively confirmed by instrumental assessment such as videofluoroscopy. Aspiration should therefore be actively considered—and, where possible, objectively evaluated—in any patient with a history of EA presenting with respiratory symptoms or recurrent lower respiratory infections, in order to prevent the development of chronic pulmonary disease [23].

Asthma represents a significant respiratory comorbidity among EA survivors, with a prevalence considerably higher than that of the general population. A retrospective cohort study demonstrated that EA patients were more than twice as likely to develop asthma compared to matched controls (OR = 2.32, 95% CI = 1.51–3.58) [24]. Nevertheless, establishing a definitive diagnosis of asthma in EA patients is inherently challenging, largely owing to the symptomatic overlap with GERD and tracheomalacia. A structured, multimodal diagnostic approach is therefore essential. Asthma should be confirmed in the presence of objective evidence of variable airflow obstruction with bronchodilator reversibility on spirometry, after alternative diagnoses have been appropriately excluded. In the present series, two patients have a documented history of recurrent episodes of wheezing. Both cases had a family history of atopic diseases (atopic dermatitis, food allergy and asthma in other family members). Respiratory function testing was performed in both cases using spirometry. Bronchial obstruction reversible with bronchodilators was demonstrated, thus confirming the diagnosis of asthma.

Bronchiectasis is one of the most serious long-term pulmonary sequelae in EA survivors. CT-based studies suggest it may affect up to 27% of this population. The available evidence points to chronic massive aspiration as the primary underlying mechanism, with recognized risk factors including gastric or colonic interposition, longstanding GERD, trisomy 21, undiagnosed TEF and broncho-esophageal fistula [8]. Although none of the patients in our series had documented bronchiectasis, this complication is clinically relevant in EA survivors with recurrent aspiration and recurrent lower respiratory tract infections.

Several diagnostic investigations can be used to evaluate the severity of respiratory conditions and identify their underlying causes. Upper airway malformations, such as laryngeal cleft, vocal cord paralysis, subglottic stenosis, tracheomalacia and TEF, can be diagnosed using laryngotracheobronchoscopy. When aspiration is suspected, a videofluoroscopic swallowing study can be performed to assess swallowing function. Sputum and bronchoalveolar lavage samples may be collected to culture opportunistic pathogens. In patients with respiratory infections, chest radiographs can help determine the extent of disease. Additionally, chest CT imaging can be used to identify complications such as atelectasis, air trapping, bronchiectasis, tracheal diverticula, vascular compression of the trachea, bronchial stenosis or cystic lesions. Spirometry is useful for diagnosing obstructive pulmonary disorders, while body plethysmography can assess restrictive lung disease [6].

A concern identified in Case 3 was the cumulative radiation exposure resulting from repeated diagnostic imaging, including multiple chest radiographs and a chest CT scan. Children with EA are particularly vulnerable in this regard, as their complex and often prolonged clinical course frequently necessitates repeated imaging studies [25]. Lung ultrasound represents a valuable radiation-free alternative for the evaluation of pulmonary consolidation, pleural effusion and other respiratory complications in this population. It has been shown to be highly accurate in the diagnosis of pneumonia in children, with sensitivity and specificity comparable to chest radiography, while avoiding ionizing radiation altogether [26]. Given these advantages, lung ultrasound should be considered as a first-line imaging tool in the follow-up of EA patients presenting with respiratory symptoms, reserving chest radiography and CT for cases where ultrasound findings are inconclusive or where detailed anatomical assessment is required.

In addition to respiratory sequelae, digestive complications are a major source of morbidity and are intimately linked to pulmonary outcomes, reflecting the aerodigestive continuum that characterizes this condition. The gastrointestinal causes of pulmonary symptoms include aspiration resulting from mucus or food retention in the esophagus, anastomotic strictures, impaired esophageal motility, congenital esophageal stenosis, swallowing-related aspiration, GER, recurrent or previously undetected fistulae and eosinophilic esophagitis [27].

Dysphagia can appear due to esophageal dysmotility, structural abnormalities, esophageal outlet obstruction and esophagitis. In older children, it is typically manifested by the sensation of food impaction, whereas in younger children, dysphagia may present with nonspecific features, including feeding difficulties, respiratory symptoms, vomiting and failure to thrive. The reported prevalence of dysphagia in patients with EA ranges from 38% to 85% [28].

All patients were diagnosed with GERD, two of whom had associated esophagitis and a hiatal hernia, and one had an esophageal diverticulum. GERD is highly prevalent following repair of EA with or without TEF. It is defined as the retrograde flow of gastric contents leading to recurrent regurgitation, failure to thrive, irritability, pyrosis, or cough. The pathogenesis of GER in this population is multifactorial, involving congenital esophageal dysmotility, incompetence of the lower esophageal sphincter, alteration of the angle of His, iatrogenic vagal nerve injury during surgery and widening of the esophageal hiatus [29]. A relationship between GER and asthma has been suggested, which is likely reciprocal; asthma predisposes individuals to GER, and GER, in turn, exacerbates asthma severity. GER may worsen asthma through two principal mechanisms. Firstly, it may involve a reflex pathway whereby acid in the esophagus stimulates vagal afferent nerves. Secondly, it may result in microaspiration of gastric content, leading to bronchoconstriction and promoting inflammation [8]. PPIs are commonly used in the treatment of GERD in children. However, in the long-term, many patients become unresponsive, with up to 40% of the patients needing antireflux surgery [30]. With surgical fundoplication, either an open Nissen procedure or laparoscopic, recurrence or persistence of the symptoms has been reported [30,31]. Long-term follow-up beyond childhood with surveillance for Barrett’s esophagus and esophageal cancer is mandatory [32].

Management and clinical response varied across the case series. Dietary modifications alone were sufficient for Cases 1 and 2, while Case 4 required the addition of PPI therapy. Case 6 responded to carob-flour-thickened milk. Cases 3 and 5, both diagnosed with reflux esophagitis, improved with daily PPI therapy. Overall, most patients improved across this spectrum of conservative-to-pharmacological management, supporting an individualized, response-guided treatment strategy.

Patients commonly present with multifactorial feeding difficulties attributable to structural abnormalities, esophageal dysmotility, inflammatory conditions such as gastroesophageal reflux disease or eosinophilic esophagitis, postoperative sequelae and behavioral or sensory-based feeding aversions [33]. These factors may culminate in malnutrition, which is associated with adverse effects on metabolic status, immune competence and cardiovascular and respiratory function [34]. This pattern is also reflected in our case series, as two patients from our cohort had an impaired nutritional status.

The significant aerodigestive morbidity associated with EA requires long-term follow-up and a multidisciplinary approach to care [6,35]. In the management of these patients, effective communication between subspecialties is essential to avoid diagnostic and therapeutic oversights. The pulmonologist, while focused on recurrent respiratory infections or chronic pulmonary disease, should consider GERD as a potential underlying contributor, given its high prevalence in this population and its well-established role in perpetuating airway inflammation and aspiration-related lung injury. Similarly, the gastroenterologist, whose primary focus may be on esophageal and foregut pathology, should not limit the clinical assessment to gastrointestinal endpoints. A careful inquiry into whether respiratory infections are accompanied by wheezing and, crucially, whether such wheezing demonstrates a response to bronchodilator therapy such as salbutamol is warranted. A positive bronchodilator response may indicate a reactive airway component requiring pulmonological input, whereas a lack of response may suggest structural or aspiration-related mechanisms. This cross-specialty awareness reflects the overlapping pathophysiology that characterizes EA and highlights the importance of an integrated approach to managing its respiratory and gastrointestinal sequelae.

**Figure 8:**
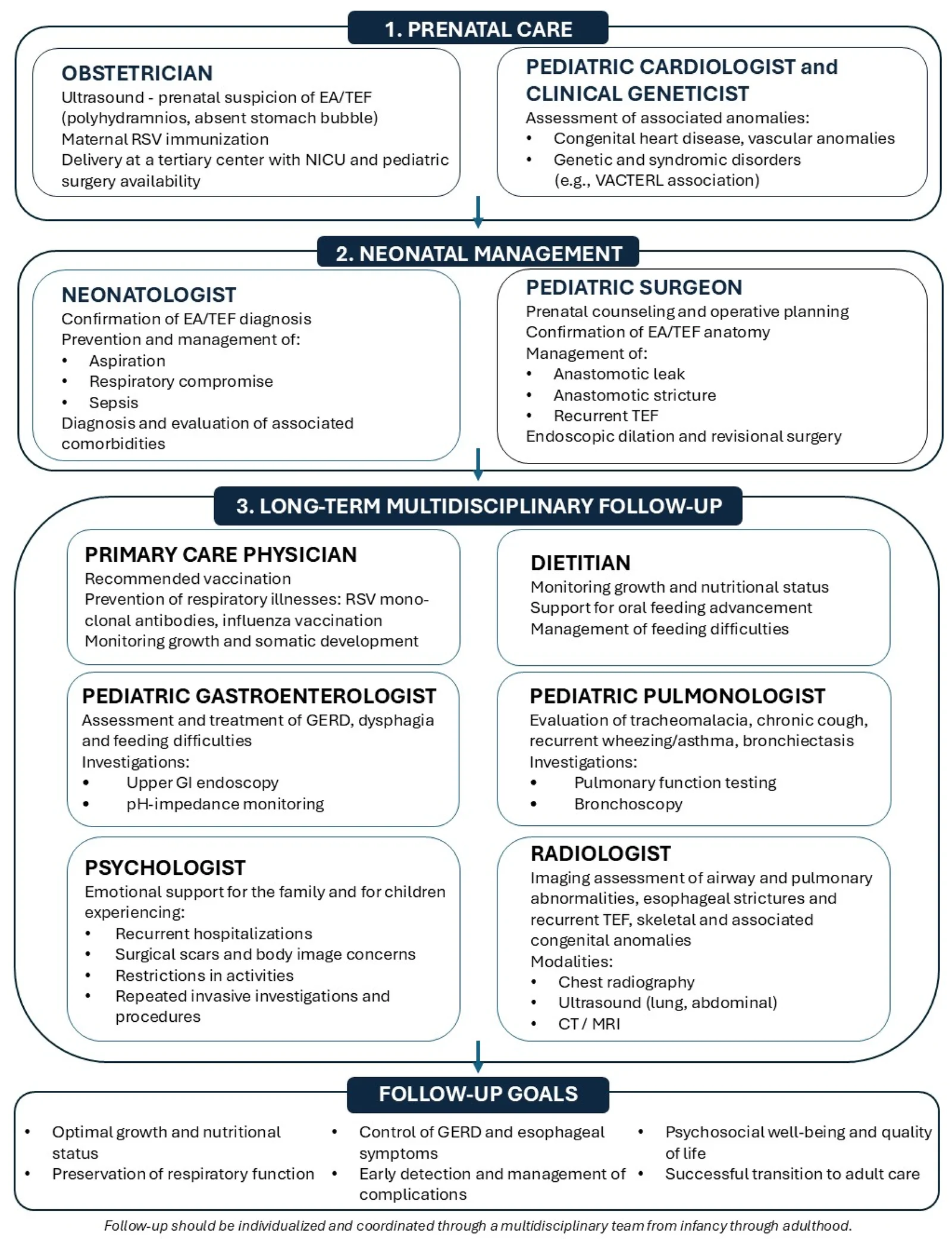
Multidisciplinary diagnostic evaluation and long-term follow-up of patients with esophageal atresia and tracheoesophageal fistula.

Based on the clinical presentations of our patients and data from the current literature, we propose a multidisciplinary follow-up algorithm for patients with EA (Figure 8).

This study has several limitations. First, it is a retrospective single-center case series with a small sample size, which limits generalizability and should be interpreted as descriptive and hypothesis-generating rather than conclusive. Second, patients were selected based on respiratory morbidity, introducing potential selection bias. Third, respiratory investigations were not uniform across all cases, and objective pulmonary function testing was available only in older children. Another limitation of this study is that aspiration was based on clinical suspicion rather than objective instrumental confirmation (e.g., videofluoroscopic swallow study) in our patients. Therefore, the reported association between aspiration and respiratory morbidity should be interpreted as a clinically inferred mechanism rather than a definitively established one. Finally, long-term follow-up data were incomplete for some patients. Thus, in Case 4, the suspicion of a possible residual or recurrent tracheoesophageal fistula had not been evaluated by bronchoscopy at the time of manuscript preparation. Case 6 was included in the cohort at 6 weeks of age, as he was one of the most recently operated-on patients admitted to our department with acute respiratory distress.

## 6. Conclusions

EA with TEF is a congenital condition with lifelong implications, frequently associated with respiratory complications. In this retrospective case series, we presented six pediatric patients with Gross type C EA with distal TEF, selected from a larger cohort based on associated respiratory morbidity, systematically detailing their clinical presentation, medical history, laboratory and imaging findings, as well as therapeutic management. Given the small, selected sample, these findings should be interpreted as illustrative rather than representative of the broader EA population. These cases illustrate the heterogeneous and multifactorial nature of respiratory and aerodigestive morbidity that can occur in children with repaired EA with distal TEF and underscore the importance of individualized diagnostic and therapeutic approaches. This case series may provide a useful reference for clinicians and contribute to the basis for future research aimed at optimizing the care of pediatric patients with repaired EA.

## Figures and Tables

**Table 1:** Respiratory complication overview.

Case	Age	Gender	Respiratory Complications							
			Tracheomalacia	Laryngitis N	Bronchiolitis N	Bronchitis N	Interstitial/Lobar Pneumonia N	Aspiration Pneumonia N	Asthma	Risk Factors
1	1 y	M	Yes		3			1 *		Passive smoking
2	2 y	M	Yes	1	4		3			Patent foramen ovale Persistent ductus arteriosus Pulmonary hypertension Passive smoking
3	10 y	F	Yes	10	40	20	7	2	Yes	Incomplete vaccination
4	6 y	M	Yes	2	1		3	1 *	Yes	Patent foramen ovale Allergic rhinitis
5	11 m	M	Yes	2			4	1 *		Selective partial IgA deficiency
6	6 w	M	Yes					1		Ventricular septal defect

y—year; m—month; w—week; M—male; F—female; N—number of episodes; *—shortly after EA surgery.

## Data Availability

The original contributions presented in this study are included in the article. Further inquiries can be directed to the corresponding author.

## Abbreviations

The following abbreviations are used in this manuscript:

AND/ASPEN	Academy of Nutrition and Dietetics/American Society for Parenteral and Enteral Nutrition
CRP	C-reactive protein
CT	computed tomography
EA	esophageal atresia
ESPGHAN/NASPGHAN	European Society for Pediatric Gastroenterology, Hepatology, and Nutrition/North American Society for Pediatric Gastroenterology, Hepatology, and Nutrition
FEV1	Forced Expiratory Volume in 1 s
GER	gastroesophageal reflux
GERD	gastroesophageal reflux disease
GINA	Global Initiative for Asthma
ICD-10	International Classification of Diseases, 10th Revision
MRI	magnetic resonance imaging
PPI	proton pump inhibitor
RSV	respiratory syncytial virus
TEF	tracheoesophageal fistula
